# Geographical and economic influences on neuroimaging modality choice

**DOI:** 10.1098/rsos.231496

**Published:** 2024-05-01

**Authors:** Niall W. Duncan, Charlotte L. Rae

**Affiliations:** ^1^ Graduate Institute of Mind, Brain and Consciousness, Taipei Medical University, Taipei, Taiwan; ^2^ School of Psychology, University of Sussex, Brighton, UK

**Keywords:** neuroimaging, global science, funding, generalizability

## Abstract

The current neuroimaging literature is unrepresentative of the world’s population due to bias towards particular types of people living in a subset of geographical locations. This is true of both the people running the research and those participating in it. These biases mean we may be missing insights into how the brain works. As neuroimaging research expands out to more of the world, the reality of global economic disparities becomes salient. With economic conditions having an effect on many background conditions for research, we can ask whether they also inﬂuence the neuroimaging research being done. To investigate this, the number of neuroimaging publications originating from a country was used as a proxy for the type of research being done there in terms of imaging modalities employed. This was then related to local economic conditions, as represented by national gross domestic product and research and development spending. National financial metrics were positively associated with neuroimaging output. The imaging modalities used were also found to be associated with local economic conditions, with magnetic resonance imaging (MRI) research positively and electroencephalography (EEG) negatively associated with national research spending. These results suggest that economic conditions may be relevant when planning how neuroimaging research can be expanded globally.

## 1. Introduction

Much of the current scientific literature on human biology and behaviour originates from a limited set of countries and demographic contexts [1]. This also applies to neuroimaging research, where various biases in participation have been noted [2–4]. Such biases, both in who is doing research and who is participating in it, may be causing us to have an incomplete or misleading understanding of the brain. This could be through participant sampling that results in non-generalizable conclusions or through the exclusion of contributions by researchers with different knowledge and perspectives [4–6]. As recognition of these issues has increased, the importance of expanding neuroimaging research to include people from more of the planet has come further into focus.

Although there are various ongoing technological developments that may lower costs, neuroimaging remains an expensive endeavour [7,8]. This presents challenges to the expansion of neuroimaging research to more parts of the world, given the profound economic disparities that exist globally. These can influence access to multiple prerequisites for doing neuroimaging, such as access to equipment, the availability of relevant training and the existence of background infrastructure like stable electricity supplies [3,9]. This in turn is likely to influence opportunities for people to contribute to the neuroimaging literature.

Since meaningful change to these economic imbalances seems unlikely in the near term, it could be advantageous to better understand how they influence current neuroimaging research. In particular, we can ask if economic conditions influence what type of neuroimaging research people do at the national level. Do local conditions have an equal influence on all imaging modalities or do they potentially guide people to work with some modalities over others?

To investigate these questions, we used the number of neuroimaging publications originating from a country, categorized by imaging modality, as a proxy for what neuroimaging is being done there. At the same time, a set of national economic metrics were taken to represent general local economic conditions within a country. These data were then combined in a set of analyses that aim to show what neuroimaging research is currently being done where, and how any differences in the modalities used may be related to economic factors.

## 2. Methods

Twenty journals were studied (table 1). These were a mixture of general brain science, general neuroimaging and neuroimaging modality specific. The selection aimed to cover a range of perceived journal prestige levels.

**Table 1.** Journals studied and the number of articles obtained from each. Articles were included in the analysis if they had both location and modality information available.

journal	total articles	included articles	proportion included (%)
*BMC Neurosci*	1431	319	22.3
*Brain Cogn*	1623	745	45.9
*Brain Stimul*	2968	1045	35.2
*Brain Topogr*	1284	845	65.8
*Cereb Cortex*	7085	3219	45.4
*Clin EEG Neurosci*	1120	635	56.7
*Cogn Neurosci*	660	193	29.2
*Eur J Neurosci*	6003	1273	21.2
*eNeuro*	2512	414	16.5
*Front Hum Neurosci*	8898	4268	48.0
*Front Neurosci*	13 131	3648	27.8
*Hum Brain Mapp*	6571	4369	66.5
*J Biomend Opt*	4937	279	5.7
*JCBFM*	4484	1288	28.7
*JMRM*	8207	4520	55.1
*J Neurosci*	17 155	3313	19.3
*Magn Reson Med*	9989	5720	57.3
* NeuroImage*	17 178	11 869	69.1
* Neurophotonics*	636	266	41.8
*Psychophysiol*	3156	1482	47.0

*JCBFM*, *Journal of Cerebral Blood Flow and Metabolism*; *JMRM*, *Journal of Magnetic Resonance in Medicine*.

### 2.1. Article information

The authors, title, abstract, keywords and medical subheading (MeSH) terms were obtained from the PubMed database (https://pubmed.ncbi.nlm.nih.gov/) for all articles published between January 2010 and June 2023 in the 20 journals studied (table 1). Searches were conducted with the Entrez API via the *biopython* tool [10]. The search terms consisted of the journal name, along with the date range. Returned articles that did not have a title, abstract and author list were excluded.

The location from which an article originated was defined as the country of the first listed affiliation of the article’s senior (i.e. last) author. Articles where the listed affiliation did not include a country were excluded. Countries were also grouped into eight geographic regions (see electronic supplementary material, table S1 for groupings). Since the choice of the author to extract a location from could influence the results, we also ran the analysis using the first author’s main affiliation. These results are presented in the electronic supplementary materials and it is noted in the main text where this choice did change the results obtained.

Titles, abstracts, keywords and MeSH terms were then searched for phrases associated with different neuroimaging modalities: electroencephalography (EEG), near infrared spectroscopy (NIRS), magnetoencephalography (MEG), magnetic resonance imaging (MRI), radiotracer imaging (positron emission tomography (PET) + single photon emission computed tomography (SPECT)), intracortical recordings and electrical/magnetic brain transcranial stimulation (see electronic supplementary material for the terms used). Multiple modalities could be marked for any single article. Studies included those based on human and non-human animals. Studies could also be based on open-access datasets, such as the Human Connectome Project [11].

Having obtained location and modality information for each article, the latter was then summed for each country. This gave a count of the number of articles published in each country that used a particular neuroimaging modality.

### 2.2. Economic information

Country gross domestic product (GDP), GDP per capita and percentage of GDP spent on research and development (R&D) were obtained from the World Bank website (https://data.worldbank.org/). Information about Taiwan was obtained from various Taiwanese government resources. GDP figures were for 2022. R&D figures were from different years, depending on the country (2014–2020). The absolute amount spent on R&D for a country was calculated from the total GDP and the percentage R&D spend. R&D information was not available for Bangladesh, Dominican Republic, Lebanon and Pakistan.

### 2.3. Statistical analysis

Whether the overall number of articles changed across years was first tested through Poisson regression, with year as an ordered categorical factor (*number of articles ∼ year*). This was done from 2014, which is the first year where all journals were active, to 2022, the last full year studied.

Changes in the proportion of articles coming from different regions over the 2014–2022 period were tested through a chi-square test. Significance was estimated through simulation due to the small counts for some regions. This was followed by adjusted standardized residuals post hoc tests to establish which regions had unexpected publication numbers in particular years [12]. Post hoc *p*-values were adjusted with false discovery rate (FDR) correction.

Regional differences in modality choice were tested through chi-square test with significance tested through simulation. This was followed by post hoc tests to establish which modalities had unexpected publication numbers in which region (FDR adjusted *p*-values).

The association of GDP per capita and R&D spending with the number of articles produced by a country was tested with a single Poisson regression where both metrics were included as predictors (*number of articles ∼ GDP per capita + R&D spending*). Overall model significance was tested through comparison with a null, intercept-only model.

Finally, modality preference was parametrized as the proportion of articles using that modality published by a country, compared with the total number of articles from that country. The relationship between modality choice and country R&D spending was then tested through mixed-effects Bayesian zero-one inflated beta regression, with country as a random eﬀect (*article proportion ∼ R&D spending* ∗ *modality +* (*1 | country*)). This approach was necessary as some countries had no articles for some modalities and all articles from other countries used a single modality, meaning the proportion distributions included both ones and zeros. R&D spending was log-transformed before being entered into the analysis. The interaction effect was tested through model comparison with a model that excluded that term.

Statistical analyses were conducted in R v. 4.3.1. Poisson regressions were conducted with the base *GLM* function, along with the base *ANOVA* function to test for main and interaction effects. Chi-square tests were conducted with the base *chisq* function and post hoc tests were conducted with the *chisq.posthoc.test* library. The *brms* package was used for beta regression [13]. Beta regression model comparison was done via the *bayestestR* package [14]. Visualization was done in Python v. 3.7.10 using *matplotlib* [15] and *geopandas* [16].

## 3. Results

### 3.1. Articles

The search returned a total of 1 21 028 articles. Of these, 1 18 960 had a title, abstract and author list (98.3%). The number of articles without this information varied between journals from 0.5% (*Human Brain Mapping*) to 33.8% (*Brain Stimulation*). As shown in table 1, the proportion of these includng articles for which both location and modality could be established ranged from 5.7% to 69.1%. The journals with low numbers of usable articles tended to be ones that were not neuroimaging specific. This gave a ﬁnal set of 49 710 articles for analysis (41.1% of the total article set). Using the first author’s location, rather than the last, reduced the number of articles with usable locations and modalities to 49 621.

Taking articles published between 2014 and 2022 (the first year where all journals existed and the last full year), we see a main effect of the year on the number published (*χ* ^2^ = 354.8, *p* = 2.2 × 10*^−1^* ^6^). This reflects a general increase in article numbers over time, with numbers going from 3580 in 2014 up to a peak of 4607 in 2021. That peak is followed by a fall to 4129 in 2022 (electronic supplementary material, figure S1).

### 3.2. Imaging modalities

**Figure 1.**
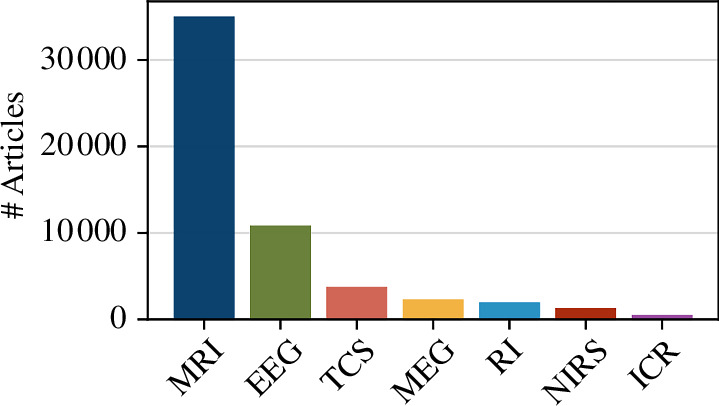
Number of articles for each modality. ICR = intracortical recordings; RI = radiotracer imaging (PET+SPECT); TCS = transcranial stimulation.

Looking at the relative popularity of each imaging modality (figure 1), MRI was found to be the most commonly used, with 35 023 articles (70.5%). The least commonly used modality was intracranial recording, being used in 538 articles (1.1%).

### 3.3. Geographical distribution

**Figure 2.**
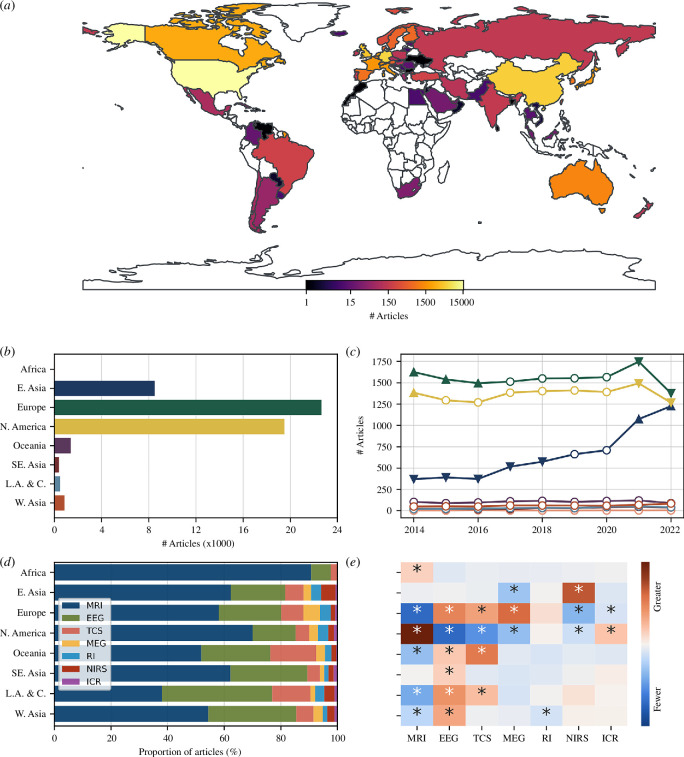
(*a*) Number of publications from each country, shown on a log scale. (*b*) Total number of publications from each region. (*c*) Yearly publications from each region. Chi-square results are indicated by markers: empty circles show no difference from expected; upward triangles show more than expected; and downward triangle show fewer than expected. (*d*) Proportion of publications per modality for each region. (*e*) Difference from expected for each modality. Colours represent standardized residuals. Asterisks indicate significant effect (*q* < 0.05). ICR = intracortical recordings; L.A. & C. = Latin America and the Caribbean; RI = radiotracer imaging (PET+SPECT); TCS = transcranial stimulation. Results using first author locations are shown in electronic supplementary material, figure S3.

Articles came from 72 different countries, with a median number of articles per country of 66.0 (interquartile range (IQR) = 8.8–477.2). Looking at the geographical distribution of articles in figure 2*a*, the majority appear to originate from Europe and North America. This is confirmed by grouping articles by region rather than country, with Europe producing the most (*n* = 20 567) and Africa the least (*n* = 43; figure 2*b*).

The contribution of different regions to the number of articles being produced each year changed over time (*χ* ^2^ = 974.92, *p* = 5.0 × 10*^−^* ^4^; figure 2*c*). Africa, Oceania, Southeast Asia, Latin America and the Caribbean and West Asia all remained relatively constant in their contributions. In contrast, East Asia showed a large increase in publications in 2020 and 2022. Europe and North America showed a relative decrease in the same period.

The proportion of articles produced with each modality differed between regions (*χ* ^2^ = 1765, *p* = 2.2 × 10*^−^* ^6^; figure 2*d*). Based on post hoc standardized residual tests (figure 2*e*), we see, for example, relatively higher numbers of MRI-based articles coming from Africa and North America; higher numbers of MEG-based articles from Europe; higher numbers of articles using EEG and transcranial stimulation (TCS) from Latin America and Caribbean; and a relatively high number of NIRS articles originating from East Asia. Similarly, we see fewer than expected MRI-based articles from Europe, Oceania, Latin America and the Caribbean and West Asia; lower numbers of EEG, TCS and MEG articles from North America; and lower numbers of radioligand-based imaging articles from West Asia. Note that there was not a significant effect for MRI studies from Africa when first author locations were used (electronic supplementary material, figure S3*a*).

### 3.4. Economic influences

**Figure 3.**
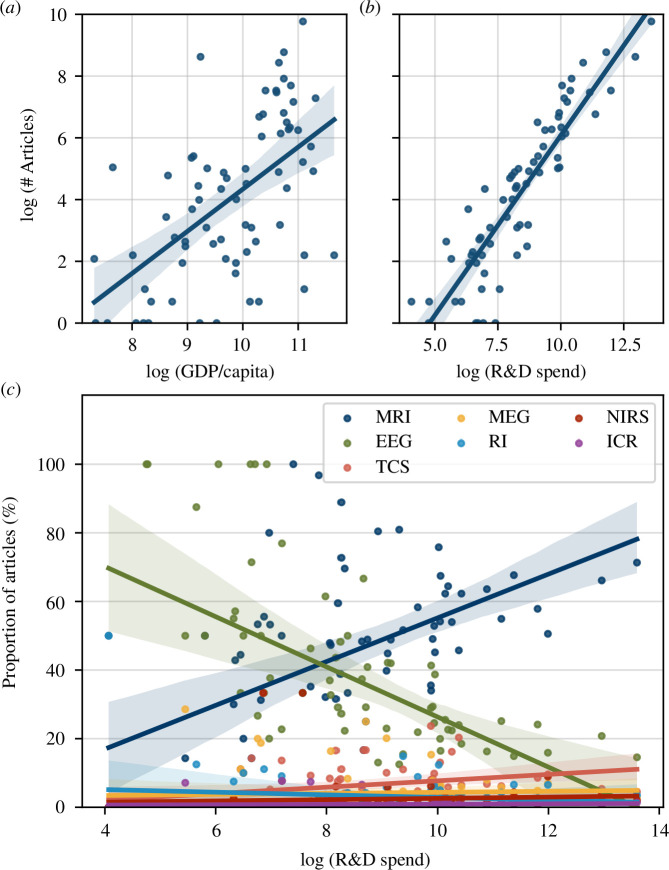
Relationship of number of articles published with GDP per capita. (*a*) and R&D spending (*b*). Values are shown on a log–log axis for visualization purposes. R&D spending is associated with the modality that tends to be used in a country (*c*). Results using first author locations are shown in electronic supplementary material, figure S4.

The number of articles produced by a country was positively associated with both the GDP per capita and the amount spent on R&D (*χ* ^2^_(65)_ = 68 044, *p* < 2.2 × 10^−16^). R&D spend was found to explain article numbers better (*β* = 4.22 × 10^−6^, *z* = 328.4, *p* < 2.2 × 10^−16^) than did GDP per capita (*β* = 1.34 × 10^−5^, *z* = 71.2, *p* < 2.2 × 10^−16^ (figure 3*a*,*b*)).

An interaction between R&D spending and modality choice was observed (Bayes factor = 1.88 × 10^6^). In line with what is seen when plotting the data (figure 3*c*), we see a positive parameter estimate for the MRI term (*β* = 0.2, 95% CI = 0.04–0.36) and a negative one for EEG (*β* = −0.21, 95% CI = −0.38 to −0.05). The remaining parameter estimate distributions were all centred close to 0, suggesting that an influence of R&D spending on them is unlikely. It should be noted, however, that the negative estimate for EEG use had less evidence when using first author locations (*β* = −0.07, 95% CI = −0.23 to 0.10).

## 4. Discussion

The results highlight the large amount of neuroimaging research that is being done around the world, with over 44 000 articles being identified from just 14 journals. The number of articles being produced has also been increasing over time, although a dip was seen in 2022 that is likely to reflect research facilities being shut down by COVID. The results also highlight how the majority of this large volume of neuroimaging research has originated from a relatively small subset of the world. Indeed, either one of Europe or North America by themselves have, since 2010, produced more neuroimaging research than the rest of the world combined. Some developments have been seen, with East Asia showing a relative increase in the number of articles being produced since 2020, bringing that region closer to parity with Europe and North America. The same increase is not, however, seen in any other under-represented region.

MRI research was seen to be the most common type of neuroimaging being done in all regions. This is consistent with its wide use in clinical research. The relative popularity of modalities is not uniform across regions though, with MEG, for example, being relatively more popular in Europe, and relatively less popular in East Asia and North America. Interestingly, we see a relatively high proportion of MRI articles coming from Africa, which may reflect recent efforts to increase access to clinical MRIs in that region; however, the overall number remains very small, and several publications discuss the need for more Africa-based MRI research, rather than reporting empirical studies [17,18]. This relatively small number may also explain why the effect is not seen when using the first author locations, as small changes in counts will have a large effect on the resulting statistics. At the same time, we see relatively high numbers of EEG and brain stimulation articles coming from Latin America and the Caribbean (along with a low number of MRI ones), perhaps reflecting that region’s long history of neurological research [19]. Together, these examples may illustrate how an interplay of historical factors and current resource flows can influence the type of research being done.

The amount of neuroimaging research being done in a country was found to be closely related to the financial resources available therein. Such a relationship is not unexpected given the need for specialized equipment and training prior to conducting neuroimaging research. This relationship may be weakened in the future as access to open neuroimaging tools, data and training increases. With these, people have the potential to conduct neuroimaging research with relatively low capital investment required. As such, investment in such open-science initiatives may be an effective way to increase global participation in neuroimaging research. It might be noted, however, that this strategy has greater potential to increase the participation of researchers from different countries than it does for changing the balance of data acquisition towards under-represented populations. Importantly, though, increasing researcher participation in this way is likely to be an important step towards achieving that latter aim.

A further potential resource barrier may arise through the need for high-performance computing to analyse neuroimaging data. This is particularly so for big datasets, which are increasingly a feature of open neuroimaging resources. Thus, even if one can access open resources, one may not be able to use them. High-performance computing requires both physical infrastructure (servers) and a stable energy supply. The increasing availability of commercial cloud-computing services may promote accessibility for those without access to institutional computing, but still requires financial outlay, and also raises questions of social and environmental sustainability [20].

Specific modality choices were also found to be related to local economic conditions. Increasing MRI research with higher R&D spending is likely to reflect the high initial cost of MRI scanners. It may also be related to the availability of MRI scanners within healthcare systems with more financial resources. In contrast, we see that EEG research is more common in countries with fewer ﬁnancial resources. This may reflect the lower cost of EEG devices. Similarly, it may also reflect a relatively higher availability of such devices in less well-funded healthcare systems.

An influence of economic conditions on modality selection may be relevant to efforts to increase the participation of currently under-represented populations in neuroimaging research. If, for example, EEG devices and expertise are more available in countries with fewer financial resources, then we may see projects target that modality in order to reduce barriers to participation. At the same time, it suggests that increased participation in more capital-intensive research types may require direct investment in local infrastructure to be feasible (along with the development of imaging devices that are not affected by factors such as skin pigmentation, hair type and culturally related dress [21–23]). This may point to a need for long-term funding mechanisms based on collective scientific goals [24].

## 5. Limitations

The methods used here have some limitations. The article search is limited to a subset of journals, which could have led to bias in the results. The journals were selected to minimize this possibility, while also ensuring that the results returned would be specific to neuroimaging, but the possibility cannot be excluded. Relatedly, locations were based on the affiliation of senior authors (and replicated with first authors). Using only one author’s location could present a misleading picture where researchers from multiple countries work on the same article. This issue could be seen within the context of existing discussions about international collaborations and the relative influence of researchers from already well-represented countries. Our economic metrics also have their limitations. GDP estimates are from single years, while publications are taken from over a decade. This could lead to some mismatch between data, should there be significant changes in GDP for a country over the period studied. A similar issue is seen for R&D spending estimates, which are also from a single year (and not the same year for all countries). It would be interesting to see more fine-grained relationships over time should relevant data be made available. Finally, the nature of our analysis means that it does not account for biases in the publication system that means work from lower-income countries (particularly those where English is not the primary language) faces more barriers to publication [25–27]. This could result in a mismatch between the amount of work actually being done in such countries and the amount that ultimately makes it to one of the journals studied.

## 6. Conclusion

The results show that while neuroimaging research is a global endeavour, participation in it is not equally distributed. Furthermore, we also see that the type of neuroimaging, and not just the volume, differs depending upon where in the world one is. These modality diﬀerences may, in turn, be related to local economic conditions. These findings point to the need for continued efforts to include more of the world in neuroimaging research and suggest that economic factors may be a relevant consideration for planning such projects.

## Data Availability

The data used in this work and the related analysis code are available at [28]. Electronic supplementary material is available online [29].

## References

[1] Henrich J , Heine SJ , Norenzayan A . 2010 The weirdest people in the world? Behav. Brain Sci. **33** , 61–83. (10.1017/S0140525X0999152X) 20550733

[2] Chen Z *et al* . 2023 Sampling inequalities affect generalization of neuroimaging-based diagnostic classifiers in psychiatry. BMC Med. **21** , 241. (10.1186/s12916-023-02941-4) 37400814 PMC10318841

[3] Geethanath S , Vaughan JT . 2019 Accessible magnetic resonance imaging: a review. J. Magn. Reson. Imaging **49** , e65–e77. (10.1002/jmri.26638) 30637891

[4] Ricard JA , Parker TC , Dhamala E , Kwasa J , Allsop A , Holmes AJ . 2023 Confronting racially exclusionary practices in the acquisition and analyses of neuroimaging data. Nat. Neurosci. **26** , 4–11. (10.1038/s41593-022-01218-y) 36564545 PMC12884511

[5] Adetula A , Forscher PS , Basnight-Brown D , Azouaghe S , IJzerman H . 2022 Psychology should generalize from — not just to — Africa. Nat. Rev. Psychol. **1** , 370–371. (10.1038/s44159-022-00070-y)

[6] Yarkoni T . 2020 The generalizability crisis. Behav. Brain Sci. **45** , e1. (10.1017/S0140525X20001685) 33342451 PMC10681374

[7] Arnold TC , Freeman CW , Litt B , Stein JM . 2023 Low-field MRI: clinical promise and challenges. J. Magn. Reson. Imaging **57** , 25–44. (10.1002/jmri.28408) 36120962 PMC9771987

[8] Frey J . 2016 Comparison of an Open-hardware Electroencephalography Amplifier with Medical Grade Device in Brain-computer Interface Applications. In Proc. of the 3rd Int. Conf., Lisbon, Portugal, pp. 105–114.

[9] Katus L *et al* . 2019 Implementing neuroimaging and eye tracking methods to assess neurocognitive development of young infants in low- and middle-income countries. Gates Open Res. **3** , 1113. (10.12688/gatesopenres.12951.2) 31508580 PMC6719506

[10] Cock PJA *et al* . 2009 Biopython: freely available Python tools for computational molecular biology and bioinformatics. Bioinformatics **25** , 1422–1423. (10.1093/bioinformatics/btp163) 19304878 PMC2682512

[11] Van Essen DC , Smith SM , Barch DM , Behrens TEJ , Yacoub E , Ugurbil K , WU-Minn HCP Consortium . 2013 The WU-Minn human connectome project: an overview. Neuroimage **80** , 62–79. (10.1016/j.neuroimage.2013.05.041) 23684880 PMC3724347

[12] Beasley TM , Schumacker RE . 1995 Multiple regression approach to analyzing contingency tables: post hoc and planned comparison procedures. J. Exp. Educ. **64** , 79–93. (10.1080/00220973.1995.9943797)

[13] Bürkner PC . 2017 brms: an R package for Bayesian multilevel models using stan. J. Stat. Softw. **80** , 1–28. (10.18637/jss.v080.i01)

[14] Makowski D , Ben-Shachar MS , Lüdecke D . 2019 bayestestR: describing effects and their uncertainty, existence and significance within the bayesian framework. J. Open Source Softw. **4** , 1541. (10.21105/joss.01541)

[15] Hunter JD . 2007 Matplotlib: a 2D graphics environment. Comput. Sci. Eng. **9** , 90–95. (10.1109/MCSE.2007.55)

[16] Jordahl K *et al* . 2020 Geopandas/Geopandas:v0.8.1. See 10.5281/zenodo.3946761

[17] Anazodo UC *et al* . 2023 A framework for advancing sustainable magnetic resonance imaging access in Africa. NMR Biomed. **36** , e4846. (10.1002/nbm.4846) 36259628

[18] Obungoloch J *et al* . 2023 On-site construction of a point-of-care low-field MRI system in Africa. NMR Biomed. **36** , e4917. (10.1002/nbm.4917) 36914258 PMC10330026

[19] Allegri RF . 2008 The pioneers of clinical neurology in South America. J. Neurol. Sci. **271** , 29–33. (10.1016/j.jns.2008.04.018) 18514739

[20] Souter NE , Lannelongue L , Samuel G , Racey C , Colling LJ , Bhagwat N , Selvan R , Rae CL . 2024 Ten recommendations for reducing the carbon footprint of research computing in human neuroimaging. Imaging Neurosci. **1** , 1–15. (10.1162/imag_a_00043) PMC12007543 40799714

[21] Choy T , Baker E , Stavropoulos K . 2022 Systemic racism in EEG research: considerations and potential solutions. Affect. Sci. **3** , 14–20. (10.1007/s42761-021-00050-0) 36042782 PMC9383002

[22] Duncan IC . 2001 The “aura” sign: an unusual cultural variant affecting MR imaging. AJR. Am. J. Roentgenol. **177** , 1487. (10.2214/ajr.177.6.1771487) 11717119

[23] Webb EK , Etter JA , Kwasa JA . 2022 Addressing racial and phenotypic bias in human neuroscience methods. Nat. Neurosci. **25** , 410–414. (10.1038/s41593-022-01046-0) 35383334 PMC9138180

[24] Rayne A *et al* . 2023 Collective action is needed to build a more just science system. Nat. Hum. Behav. **7** , 1034–1037. (10.1038/s41562-023-01635-4) 37291438

[25] Amano T *et al* . 2023 The manifold costs of being a non-native English speaker in science. PLoS Biol. **21** , e3002184. (10.1371/journal.pbio.3002184) 37463136 PMC10353817

[26] Murray D , Siler K , Larivière V , Chan WM , Collings AM , Raymond J , Sugimoto CR . 2019 Author-reviewer Homophily in peer review. bioRxiv.

[27] Chawla DS . 2023 Preprints become papers less often when the authors are from lower-income countries. Nature (10.1038/d41586-023-02216-1) 37400638

[28] Duncan NW , Rae C . 2024 Geographical and economic influences on neuroimaging modality choice. OSF. (10.17605/OSF.IO/6XY2Q) PMC11061638 38699551

[29] Duncan NW , Rae CL . 2024 Data from: Geographical and economic influences on neuroimaging modality choice. Figshare. (10.6084/m9.figshare.c.7100058.v1) PMC11061638 38699551

